# Engineering a Brain Cancer Chip for High-throughput Drug Screening

**DOI:** 10.1038/srep25062

**Published:** 2016-05-06

**Authors:** Yantao Fan, Duong Thanh Nguyen, Yasemin Akay, Feng Xu, Metin Akay

**Affiliations:** 1 Department of Biomedical Engineering, University of Houston, 3605 Cullen Blvd, Room 2027, Houston, TX, USA; 2 Bioinspired Engineering and Biomechanics Center, Xi’an Jiaotong University, Xi’an 710049, China

## Abstract

Glioblastoma multiforme (GBM) is the most common and malignant of all human primary brain cancers, in which drug treatment is still one of the most effective treatments. However, existing drug discovery and development methods rely on the use of conventional two-dimensional (2D) cell cultures, which have been proven to be poor representatives of native physiology. Here, we developed a novel three-dimensional (3D) brain cancer chip composed of photo-polymerizable poly(ethylene) glycol diacrylate (PEGDA) hydrogel for drug screening. This chip can be produced after a few seconds of photolithography and requires no silicon wafer, replica molding, and plasma bonding like microfluidic devices made of poly(dimethylsiloxane) (PDMS). We then cultured glioblastoma cells (U87), which formed 3D brain cancer tissues on the chip, and used the GBM chip to perform combinatorial treatment of Pitavastatin and Irinotecan. The results indicate that this chip is capable of high-throughput GBM cancer spheroids formation, multiple-simultaneous drug administration, and a massive parallel testing of drug response. Our approach is easily reproducible, and this chip has the potential to be a powerful platform in cases such as high-throughput drug screening and prolonged drug release. The chip is also commercially promising for other clinical applications, including 3D cell culture and micro-scale tissue engineering.

Brain cancer is a serious health and social issue. According to the American Cancer Society^1^, a brain cancer will be diagnosed in almost 23,000 adults, while 15,300 adults will die from it in the United States in 2016. Brain cancers cause about 7% of cancer-related deaths for those under the age of 70. For children and teens, brain cancer is the second most common form of cancer (after leukemia) and causes the most cancer-related deaths. About 4,300 children and teens will be diagnosed with a brain cancer in 2015 and more than half of them will be younger than 15 years of age^1^. Of the brain cancers, glioblastoma multiforme (GBM) is the most common and malignant of all human brain cancers, with a median survival rate of 12–15 months^2^,^3^,^4^. Currently, drug administration is one of the most effective treatments for brain cancers, which require high-throughput drug screening methods. Beside that, the promise of personalized medicine is to find the optimal drug combination for individual patients despite the vast selection of available drugs and high heterogeneity of patients. Its success relies on the rapid, chemo-sensitive screening of a particular patient. Cell arrays are widely used in biomedical fields, especially for drug screening applications^5^,^6^,^7^. However, most existing cell array technologies are based on two-dimensional (2D) cell cultures, which do not recapitulate the native *in vivo* microenvironment. In comparison, three-dimensional (3D) tissue models offer the advantages of cell-cell/cell-matrix interactions^8^,^9^ and spatial and physicochemical diversity^10^. Also, they provide a sustainable, high-throughput 3D tissue formation platform, which can be used for drug screening^11^,^12^,^13^. Therefore, the emerging tissue- and organ-on-chip concept can potentially solve current challenges in personalized drug screening.

Current cell array platforms for drug screening are constructed using microfluidic channels made from poly(dimethylsiloxane) (PDMS)^14^,^15^. The drugs flow through the microfluidic channels to compartmentalized cultured cells in parallel with spatio- temporal gradients^16^,^17^,^18^. However, the structure of these microfluidic devices is generally complicated; a representative device is the “lung-on-a-chip”^19^ that recapitulates the alveolar-capillary barrier in a lung by co-culturing human alveolar epithelial cells and human pulmonary microvascular endothelial cells in 3D engineered microfluidic chambers and channels. There are several limitations associated with the use of PDMS in these microfluidic devices^20^,^21^, such as the requirement of expensive silicon molds and a cleanroom, time-consuming and labor-intensive replica-molding from a silicon wafer, and the need for specific assembly by oxygen-plasma bonding. Also, the mechanical properties, water content, and biomolecular diffusion of PDMS is different from the native extracellular matrix (ECM). These limitations prevent PDMS microfluidic devices from mimicking the *in vivo* cellular microenvironment.

Poly(ethylene) glycol diacrylate (PEGDA) hydrogel has similar mechanical properties and water content to natural ECM. PEGDA is photo-polymerizable, so it can be easily and quickly solidified after seconds of ultraviolet (UV) exposure. PEGDA microfluidic hydrogels have been widely used for cell encapsulation because they are permeable to substances such as water, biomolecules, and chemicals^22^,^23^, and can also entrap and release drugs through diffusion^24^,^25^. These properties promise a physiologically relevant microenvironment with high spatiotemporal precision within a PEGDA hydrogel microfluidic device^11^,^26^. However, the controlled release of multiple drugs at different concentrations poses a challenge for existing microfluidic devices, especially for high-throughput drug screenings^26^.

Therefore, in this study, a novel brain cancer chip was developed using PEGDA hydrogels for drug screening by integrating a microwell array with microfluidic channels. GBM cells were cultured in the microwell array to form 3D brain cancer tissues and combinatorial treatment of Pitavastatin and Irinotecan was performed in this chip to demonstrate system advantages. The setup created an ECM-like cellular microenvironment for 3D *in vitro* culture, with a massive-parallel processing capability and a tunable transport property. The brain cancer chip platform developed here provides a tunable drug release, since a single administration of a drug can be released at a desired concentration within the microenvironment over an extended period of time.

## Results

### Brain cancer chip design and preparation

**Figure 1:**
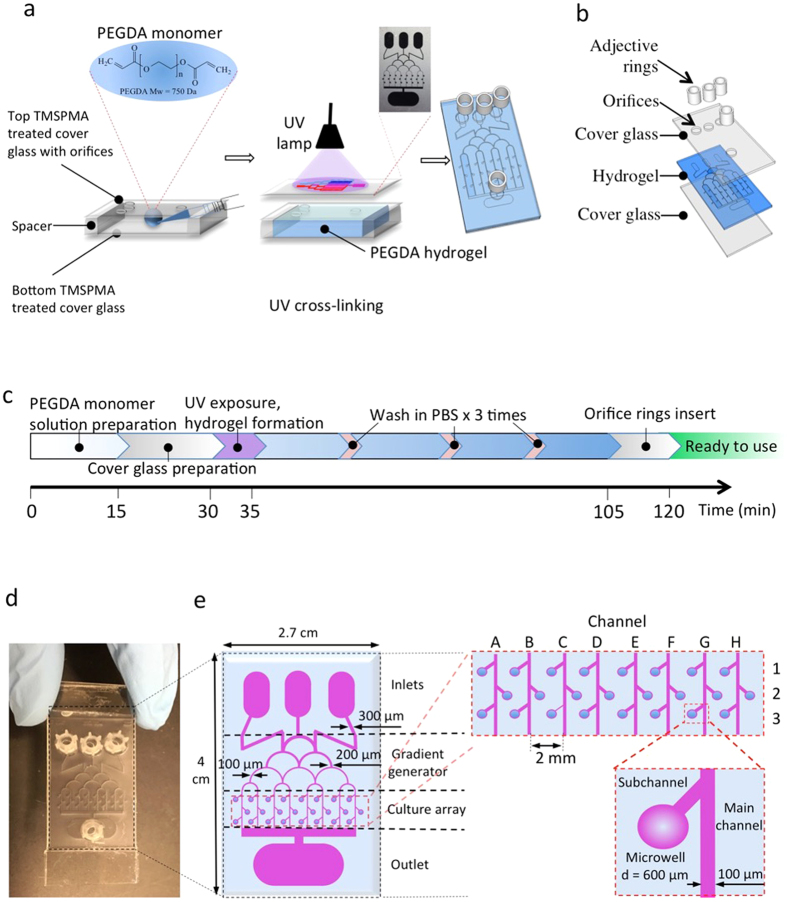
Brain cancer chip design and preparation. (**a**) Final hydrogel device with microchannels and microwells after adding inlet and outlet reservoirs on top of the inlet and outlet orifices. (**b**) Schematic of the layers that are assembled during device fabrication. (**c**) Time protocol of the brain cancer chip preparation. (**d**) Photograph of the device from above. (**e**) Christmas tree-shaped channel system (gradient generator) of the brain cancer chip with channels of gradually decreasing width from 300 μm to 100 μm, an array of 24 individual culture chambers, and three inlet reservoirs and one outlet reservoir. The sub-channels, which link the microwells to the main channel, prevented captured cells from escaping the microwell.

In this study, a brain cancer chip was developed, consisting of four reservoirs (three inlets and one outlet), top cover glass, PEGDA hydrogel layer, and bottom cover glass (Fig. 1a,b). Using a photo-polymerizable PEGDA hydrogel, this brain cancer chip was built by a simple one-step UV lithography process, as detailed in the “Methods” section. A laser-printed plastic film with designed microfluidic patterns was used for the photomask, which required a very low UV density. This technique is more cost-effective than PDMS microfluidic devices, which use more expensive, chrome photomasks. After a few seconds of UV exposure, the microfluidic structures were precisely duplicated in the PEGDA hydrogel layer from the photomask (Fig. 1a). The thickness of the PEGDA hydrogel layer was determined by the height of the spacers on the bottom cover glass, which can be adjusted from micrometer scale to millimeter scale.

Figure 1d shows the fabricated brain cancer chip with a hydrogel microfluidic dimension of 2.7 cm × 4 cm. The PEGDA hydrogel had a Christmas tree-shaped channel system (gradient generator), an array of 24 individual culture chambers (A1-3, B1-3, C1-3, *ect*.), and four reservoirs, including three inlets and one outlet (Fig. 1e). The width of the Christmas tree-shaped channels decreased gradually from 300 μm to 100 μm. In summary, the culture array had 24 culture chambers, comprised of the main channel, a sub-channel and a microwell (Fig. 1e). The sub-channels, which linked the microwells to the main channel, prevented captured cells from escaping the microwell. Based on the optimization from our previous work^26^, the constructed microwells had a diameter of 600 μm and the main channels and sub-channels had a width of 100 μm.

### Characterization of the chip

**Figure 2:**
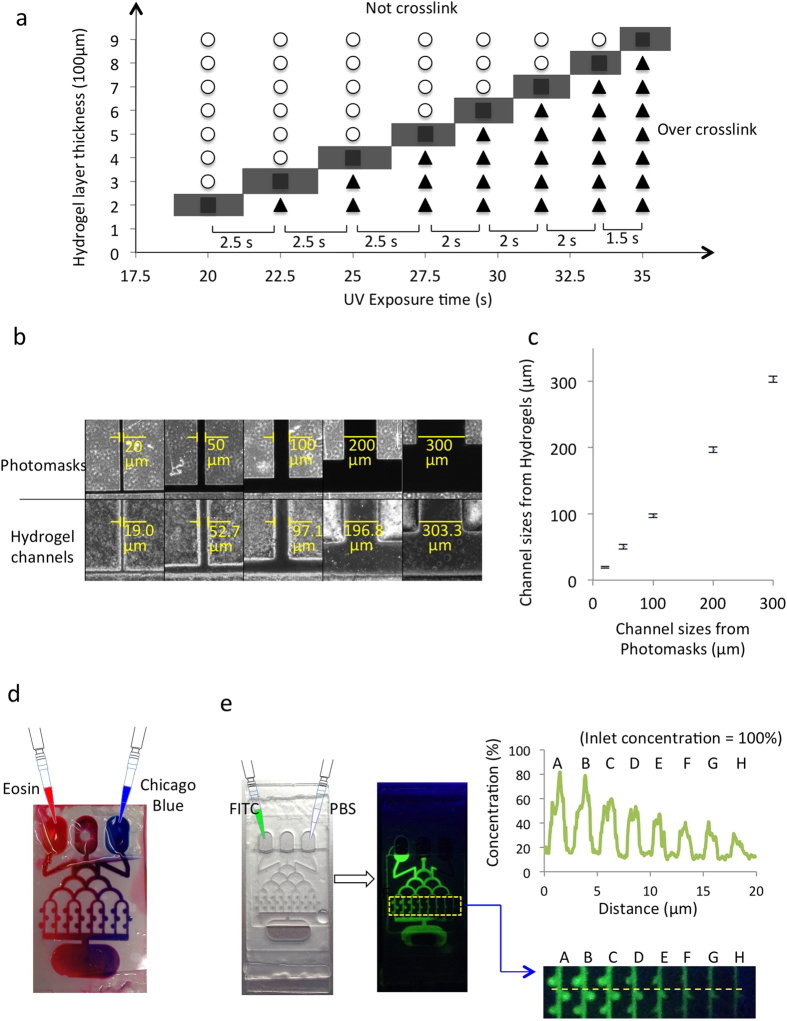
Characterization of the brain cancer chip. (**a**) Diagram for the optimization of PEGDA hydrogel layer thickness and UV exposure time for cross-linking PEGDA. Circles indicate exposure times that did not result in fully cross-linked PEGDA hydrogel, triangles indicate over-cross-linking in which the channels were blocked, and squares indicate the optimal time for crosslinking PEGDA hydrogel. (**b**) Images of photomasks with different channel widths and their resulting hydrogels. Channels were observed from top view. (**c**) Quantitative results of channel widths produced in the PEGDA hydrogel versus their corresponding photomasks. (**d**) Combinatorial screening of solutes from inlets via microfluidic networks. Eosin (red) and Chicago blue (blue) were injected into the two outer inlets. Color in the microchannels changed from red (left) to blue (right). (**e**) Fluorescent imaging of FITC in hydrogel microfluidic channels after simultaneous injection of FITC (left) and PBS (right). The concentrations of FITC solution in different microchannels were calculated by comparing fluorescent intensities between each microchannel and the inlet.

The depths of the PEGDA hydrogel microwells and channels were affected by the UV exposure time. Insufficient exposure time was found to fail to cross-link the PEGDA hydrogel, while extended exposure time resulted in over-cross-linking, which changed the dimensions of the microwells and channels. Thus, the UV exposure time was optimized for a hydrogel layer of different thicknesses by measuring the difference between the channel in the photomask and the hydrogel (Fig. 2a). For hydrogel thicknesses of 300 μm, 400 μm, 500 μm, 600 μm, 700 μm, and 800 μm, the optimal UV exposure times were 22.5, 25, 27.5, 29.5, 31.5 and 33.5 seconds, respectively. Thicker hydrogels required longer exposure times, but at decreased rates of change in UV exposure time between hydrogel thicknesses. For example, the 900 μm-thick hydrogel required 35 seconds to cross-link the PEGDA network, which is 1.5 seconds longer than required for the 800 μm-thick layer. As mentioned above, a photomask with 600 μm-diameter microwells and 100 μm-wide main channels and sub-channels was used. For a microwell thickness of 600 μm, a UV exposure time of 29.5 seconds at 100 mW/cm^2^ was optimal. The resulting hydrogel had 600 μm-diameter microwells, 100 μm-wide main channels and sub-channels, and 600 μm-deep and 200 μm-deep main channels and sub-channels, respectively.

To further quantify the spatial resolution of PEGDA hydrogel, photomasks were designed with 20, 50, 100, 200, and 300 μm-wide channels (Fig. 2b). After cross-linking, the channel widths of the PEGDA hydrogel were measured and it was found that the channels were 19.0 ± 1.27, 52.7 ± 3.39, 97.1 ± 2.59, 196.8 ± 4.16, and 303.3 ± 4.76 μm-wide, respectively (Fig. 2c). The difference between channel widths of the PEGDA hydrogel and the photomask was 3.9 ± 2.50%, and the maximum achievable resolution level in our approach was the 20-μm-wide channel.

After the chip was fabricated, its flow capabilities and leakage were assessed through exposure to dyes. Two dyes, red (Eosin) and blue (Chicago blue), were concurrently inserted into two inlets and a mixture of colors was observed to distribute through the parallel channels (Fig. 2d), indicating that no leakage or blockage occurred in the chip. It was observed that the dyes in the middle channels were mixed to the highest degree and the dyes in the outer channels were the most pure in color (red in the left and blue in the right), indicating that the Eosin concentration decreased from the left to the right channel. To further quantify the generated gradient, two 200-μl solutions of 10-μM fluorescein isothiocyanate (FITC) and phosphate-buffered saline (PBS) were simultaneously pipetted into the left and right inlets, respectively (Fig. 2e). FITC’s fluorescent intensities in the channels were measured and compared to the original solution in the inlet. The fluorescent intensity of FITC was strongest in the left channel (channel A - approximately 80%), further confirming that the solution mixture in this channel came mostly from the left inlet and the FITC concentration decreased gradually from channel A to channel H. The experimental data is similar to the computational calculation (see Supplementary Fig. S1).

### Measurement of the diffusion in the hydrogel chip

**Figure 3:**
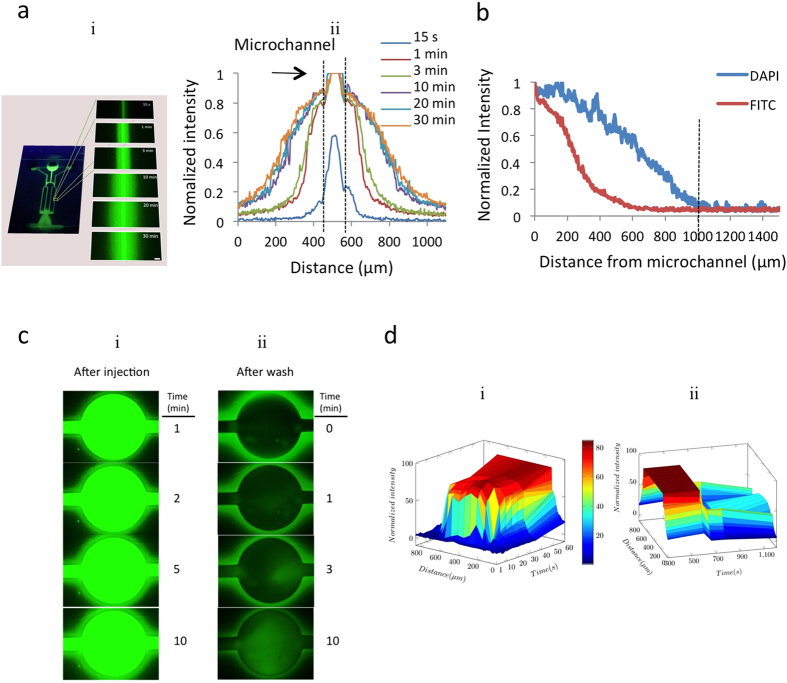
Diffusion properties of the hydrogel chip. (**a**) Diffusion of FITC-glucose in the microchannel at the time points: 15 seconds, 1 min, 3 min, 10 min, 20 min and 30 min. Scale bar, 500 μm. Plot of normalized fluorescent intensity of FITC solution for each time point. (**b**) Graph showing the diffusion of FITC-glucose (MW = 150,000 Da) and DAPI (MW = 277 Da) 30 minutes after injection. (**c**) Time-lapse images of FITC diffusion in microwell (i) and tunable release of FITC after washing microwell by PBS (ii). (**d**) Three-dimensional graph showing the increase in fluorescent intensity of the microwell and its surrounding hydrogel diagonally across the microwell over time after injection (i) and the change in fluorescent intensity of the microwell and its surrounding hydrogel after washing with PBS, showing the release of chemicals back into microwell.

PEGDA hydrogel is permeable to various substances (*e.g.*, water, biomolecules, and chemicals) and can controllably facilitate a smart release of chemicals on this chip. Chemicals, such as drugs, can be concentrated and entrapped within the hydrogel and released through diffusion over a prolonged period. To quantify the diffusion characteristics of the PEGDA hydrogel, two dyes were tested with different mass transport properties. First, the fluorescent intensity of FITC (MW = 150,000 Da) was used to measure the diffusion efficiency of the platform (Fig. 3a). Initially, the distribution of fluorescent dye in the channel was measured after diffusing 200 μl of 10-μM FITC solution from the center inlet. It was observed that FITC began to diffuse into the hydrogel after filling the channel, and reached equilibrium after 10 minutes. A similar experiment was performed with low molecular weight 4′,6-diamidino-2-phenylindole (DAPI, MW = 277 Da) to confirm the diffusional transport of low molecular weight solutes (Fig. 3b). When the distance between channels was 1 mm, the normalized intensity of both DAPI and FITC decreased to less than 4.2%.

Notably, this diffusion was driven by concentration gradient. To visualize this process, 10 μM FITC were loaded into the hydrogel microfluidic chip (see Supplementary Video 1). FITC was observed to diffuse into the hydrogel around the microwell, as shown in Fig. 3c(i), and reach equilibrium after 10 minutes. Then, 1 ml of PBS was loaded into the microfluidic inlet to thoroughly wash non-absorbed FITC from the microwell (see Supplementary Video 2). Figure 3c(ii) shows that, after washing, the fluorescent intensity of FITC increased inside the microwell and reduced in the hydrogel around the microwell. Figure 3d graphically represents the change in fluorescent intensity in the microwell after injecting FITC (Fig. 3c(i)) and after washing with PBS (Fig. 3c(ii)). After the FITC injection, the fluorescent intensity in the microwell increased to 85%. After washing with PBS, the fluorescent intensity of the microwell wall decreased to approximately 45% and the fluorescent intensity of the microwell cavity decreased to almost 0%. Ten minutes after washing, the fluorescent intensity of the microwell cavity increased to 50% (Fig. 3d), quantitatively suggesting that FITC diffused from the hydrogel walls back into the cavity of the microwell.

### GBM cell capture, culture and 3D spheroid formation

**Figure 4:**
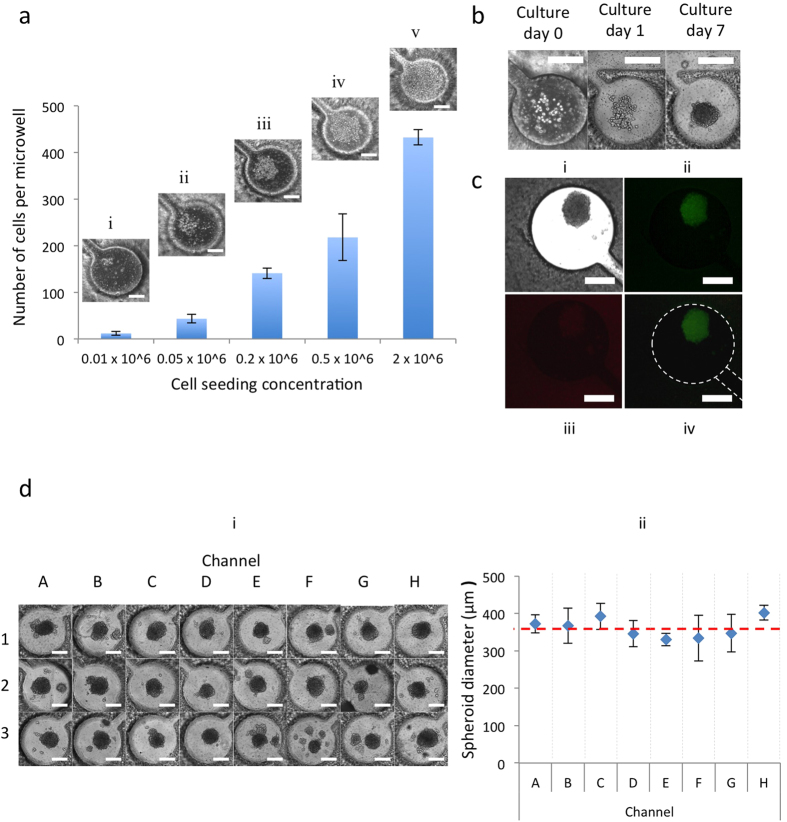
GBM cell capture, culture and 3D-spheroid formation. (**a**) Optimization of initial cell-seeding concentrations. Cells in each microwell were counted 30 minutes after the injection. Error bars represent standard error of the mean. Scale bar, 200 μm. (**b**) U87 cells were injected into the microfluidic network from the inlets and captured in the microwell. Cells were cultured in DMEM and aggregated to form cancer spheroids. Scale bar, 200 μm. (**c**) Bright field and fluorescence micrograph showing cancer spheroid. (ii) Staining of live cells with 4 mM Calcein AM (green) and (iii) dead cells with 2 mM ethidium homodimer-1 (red). (iv) Merged image of stained cancer spheroid. (**d**) GBM spheroids in microwells of brain cancer chip (i) showed a uniform size (361.3 ± 36.2 μm-diameter) (ii). Scale bar, 200 μm.

Cell capture is critical to platform efficiency, where microfluidic geometry significantly affects the cell capture efficiency. Six different microfluidic structures were designed and tested in our experiments (see Supplementary Fig. S3 and Supplementary Video 3) and the current culture chamber structure was chosen. To find an appropriate cell seeding concentration, a series of cell concentrations was assessed by using GBM cells (U87) at the following concentrations: 0.01 × 10^6 ^cells/ml, 0.05 × 10^6 ^cells/ml, 0.2 × 10^6 ^cells/ml, 0.5 × 10^6 ^cells/ml and 2 × 10^6 ^cells/ml, shown in Fig. 4a. Initially, cell concentrations of 0.5 × 10^6 ^cells/ml and 2 × 10^6 ^cells/ml were used. It was observed that the microwells were over-filled with cells under both cell concentrations in one week (Fig. 4a(iv,v)). The concentration of 0.01 × 10^6 ^cells/ml, which equals a cell density of only 42 cells/mm^2^ (around 12 cells per microwell), was too low to promote the quick formation of cell spheroids in one week (Fig. 4a(i)). For cell concentrations of 0.05 × 10^6 ^cells/ml and 0.2 × 10^6 ^cells/ml, approximately 43 and 141 cells per microwell were captured, respectively (Fig. 4a(ii,iii)). An optimal cell seeding concentration of 0.05 × 10^6 ^cells/ml was chosen.

The captured GBM cells were then cultured in the PEGDA hydrogel microfluidic chip and their development into 3D GBM spheroids in the microwells was observed (Fig. 4b). The captured GBM cells did not adhere to the channel surface because of PEGDA hydrogel’s cell-repellant properties, and they started to aggregate in the microwells after 1–2 days. The cells proliferated within the PEGDA microwells during the following culture and formed cancer spheroids with a diameter of 300–400 μm that were used after 7 days for drug testing. Because PEGDA hydrogel is biocompatible, GBM spheroids were highly viable on day 7, as shown by live/dead staining (Fig. 4c). On day 7, 24 GBM spheroids had formed in the PEGDA microwells (Fig. 4d(i)), with a uniform size (361.3 ± 36.2 μm in diameter) (Fig. 4d(ii)). The layered structure of the 3D GBM spheroid resulted in a hypoxic center similar to the *in vivo* environment of a GBM tumor mass, most common during the early stage of tumor development.

### Drug screening

**Figure 5:**
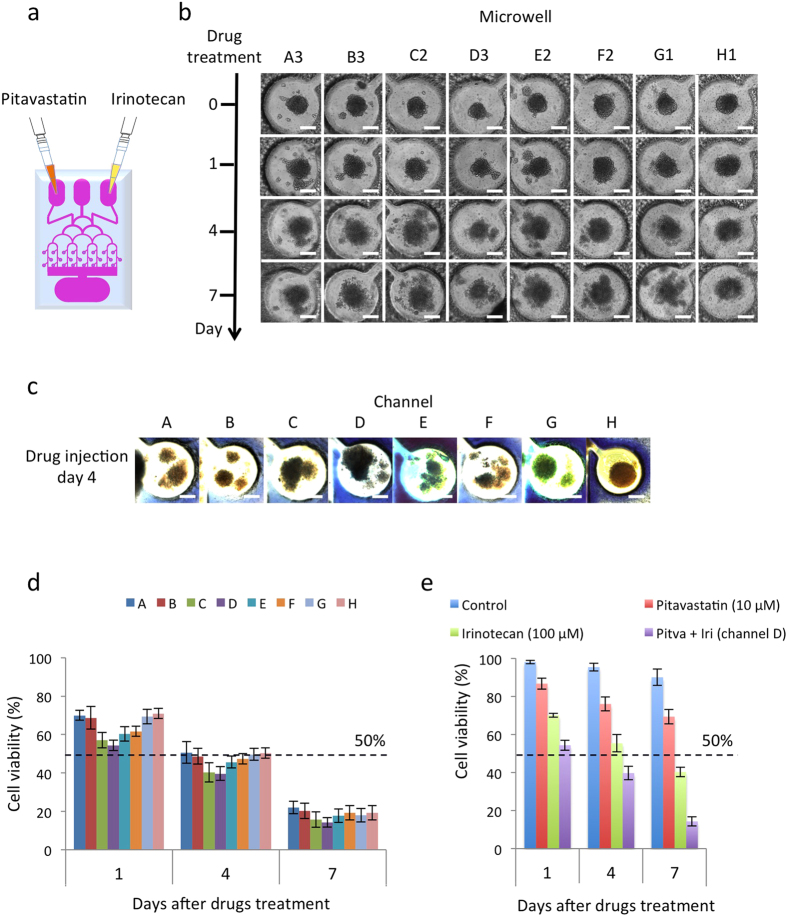
Combinatorial drug treatment and drug screening. (**a**) Pitavastatin (10 μM, 100 μl) and Irinotecan (100 μM, 100 μl) were injected into the two outer inlets on day 7 of cancer spheroid culture. (**b**) Time-lapse images of cancer spheroid in microwells were taken before drug administration (day 0) and after drug administration (day 1, day 4 and day 7). Scale bar, 200 μm. (**c**) Images of trypan blue staining of each channel on day 4 after drug administration. Scale bar, 200 μm. (**d**) Combinatorial drug screening results. Cancer spheroids were cultured for 7 days. Pitavastatin (10 μM, 100 μl) and Irinotecan (100 μM, 100 μl) were then injected into the two outer inlets. The graph shows cell viability in the microwells on day 1, day 4 and day 7 after dual-drugs treatment. (**e**) Comparison of combinatorial drug screening results (Channel D) with individual drug screening results by Pitavastatin (10 μM, 100 μl) and Irinotecan (100 μM, 100 μl) and untreated GBM cancer spheroids on day 1, day 4 and day 7 after dual-drug and individual drug treatments.

To demonstrate the effectiveness of two widely used anti-cancer drugs on GBM spheroids by the chip, two FDA approved drugs were used: Pitavastatin (trade name Livalo) and Irinotecan. Pitavastatin is a member of the statin class of drugs that inhibits the growth of several cancer cell types by suppressing inflammation, inducing apoptosis, and modulating angiogenesis^27^. Irinotecan is a topoisomerase I enzyme inhibitor that blocks topoisomerase I to prevent cells from dividing and growing^28^. These two anticancer drugs, Pitavastatin (10 μM, 100 μl)^29^ and Irinotecan (100 μM, 100 μl)^30^ were concurrently administered into the hydrogel microfluidic device via the two inlets (Fig. 5a). Figure 5b shows the effect of dual-drug administration on the cancer spheroids over time as they collapsed. The edges of the cancer spheroids were crisp and the spheroids continued to grow prior to adding the drugs. However, the structure of the cancer spheroids became loose and cells were clearly observed to detach from the spheroids, as well as undergo autophagic cell death after the drugs were added.

To assess the efficacy of different drug combinations, trypan blue dye was loaded into middle inlet of the chip to indicate cell viability. Semi-quantitative evaluation of the combinatorial drug effect for each channel was performed through observation with the naked eye. Figure 5c shows an image of the trypan blue staining for each channel. Channel D showed the most trypan blue staining, providing a quick and semi-quantitative conclusion that the concentrations of Pitavastatin and Irinotecan in channel D were superior to the concentrations in the other channels. The quantitative results of cell viability are shown in Fig. 5d (See “Methods” for more details). On day 1 of dual-drug administration, the cell viability in channel A was around 70%, while the cell viability in channel H was 71%. At that time, channel D had the lowest cell viability of approximately 55%. The cell viabilities reduced to 50% in all channels after 4 days of dual-drug administration. For channel D, the cell viability was 39.67%. The combinatorial drug treatment in channel D was also compared with the individual drug screening of Pitavastatin (10 μM, 100 μl) and Irinotecan (100 μM, 100 μl), as well as the untreated GBM spheroid cells on day 1, 4 and 7, as shown in Fig. 5e. In the untreated GBM cells, the cell viabilities were 95% on day 4 and 90% on day 7. The treatment with Pitavastatin reduced the cell viabilities to 90% on day 1, 85% on day 4 and 75% on day 7. However, the treatment with Irinotecan reduced the cell viabilities to 70% on day 1, 50% on day 4 and 40% on day 7.

In addition to the determination of approximate concentration of Pitavastatin and Irinotecan at each point using the COMSOL model (www.comsol.com), and the effect of combinatorial drug on the spheroid cell viability in our platform, we also used the Chou-Talalay (CT) predictive model (www.combosyn.com) to study the synergy and antagonism between these two drugs^31^. The experimental data, including the drug concentration and cell viability information, were entered into the CT model to predict the viability information related to the effect of combinatorial drugs on the GBM spheroid cells. Supplementary Fig. S2 shows the dose effect curve for each drug individually and their combination. The CT model also suggested that the drug combination is much more effective than individual drugs on the cell viability of the GBM cancer spheroids. Furthermore, these findings confirm the reliability of the data obtained in this study and the efficacy of using combinatorial drugs for the treatment of cancer cells.

### Tunable release of drugs

**Figure 6:**
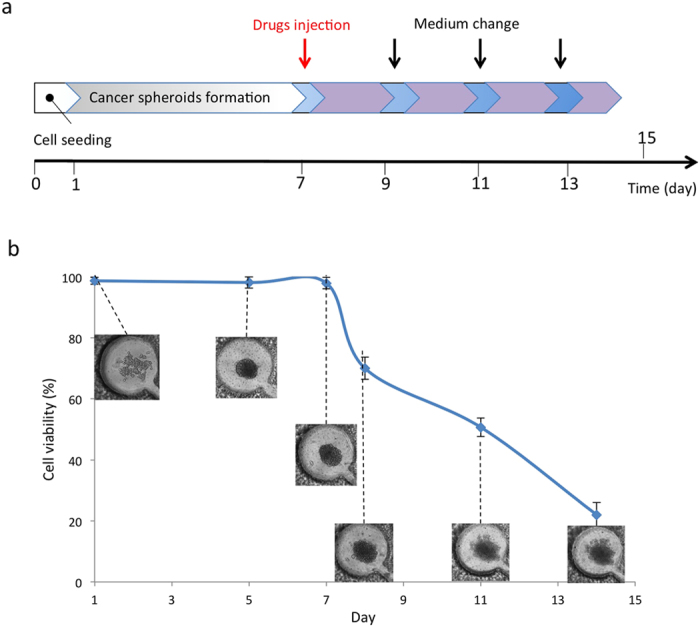
Tunable release of drugs. (**a**) Time protocol of the formation of cancer spheroids and drugs testing in hydrogel chip. (**b**) Time-lapse images and cell viability of U87 cells in microwell during the application of a single dose of drugs on day 7. Drugs were continuously released from the hydrogel layer to microwell and affected cancer spheroid.

The chip was assessed for tunable drug-release capabilities. Diffusion was observed when the drugs were applied to the GBM spheroids in the brain cancer chip. As discussed previously, we cultured 3D GBM spheroids in the hydrogel microfluidic chip and took time-lapse images of cancer spheroids in the microwells to observe the effectiveness of Pitavastatin and Irinotecan. A single administration of Pitavastatin (10 μM, 100 μl) and Irinotecan (100 μM, 100 μl) was applied to the two inlets on day 7 of cell culture (Fig. 6a). On days 8, 10, and 12, the media was replaced with fresh, drug-free culture media (Fig. 6a). Despite a single application of drugs and frequent media changes, consistent cell death in the microwells was observed over the course of the experiment (Fig. 6b). This consistent drug effect came from the drugs’ release from the hydrogel of the microwell, which was in accordance with the phenomenon seen in the FITC experiment (Fig. 3c,d). After 10 minutes, the drugs were estimated to diffuse back into the microwell. The drug concentration in the microwell stabilized once the diffused drugs reached equilibrium. Uptake of the drugs by cells in the cancer spheroids reduced the drug concentration in the microwell. Concurrently, the drug concentration reduction was counterbalanced by the equilibrium-driven drug diffusion from the hydrogel back into the microwell. This resulted in continual drug release after a single dose.

## Discussion

In this study, we developed a simple, flexible and cost-effective hydrogel microfluidic device (*i.e.*, brain cancer chip) for high-throughput drug screening based on 3D GBM spheroid culture. Our hydrogel microfluidic device was tested as a screening platform for drug testing of GBM. The results indicate that this brain cancer chip features high-throughput GBM cancer-spheroid formation by providing a large amount of micro-sized culture chambers, multiple-simultaneous drug administration (that can be economically modified and flexibly constructed by re-designing the microfluidic patterns), and massive-parallel testing of drug response under a biocompatible 3D microenvironment. This chip perfectly fills the gap for a much-needed screening tool to personalize medicines. As an *in vitro* 3D cell culture model, this chip could help reduce the time and cost to perform preclinical studies. The proposed novel platform could be useful and cost-effective for high-throughput screening of cancer drugs and assessment of treatment responses in personalized medicine.

In comparison to the normal fabrication of PDMS microfluidic devices that usually takes two days, the fabrication of our PEGDA hydrogel microfluidic chip takes less than two hours, which is more time-efficient (Fig. 1c). While measuring the diffusion in the hydrogel chip, it was noted that at an inter-channel distance of 1 mm, the normalized intensity decreased to less than 4.2%, as seen in Fig. 3b. This result suggests that cross talk between channels in the chip could be eliminated by increasing the distance between each channel to over 2 mm. Upon visualization of the diffusion process, the diffusion was observed to be driven by the concentration gradient, which clearly indicates that this diffusion process is tunable.

Following dual-drug administration, the cell viability in each of the channels was assessed and channel D was determined to have the lowest level of cell viability of approximately 55% on day 1 of dual-drug administration (Fig. 5d), suggesting that the most effective combinatorial treatment of Pitavastatin and Irinotecan came from channel D, where the concentration of Pitavastatin was 6 μM and Irinotecan was 40 μM, according to the previous quantitative characterization (Fig. 2e(iii)). Also, the cell viabilities in all channels decreased to below 50% after 4 days of dual-drug administration, and the cell viability at that time in channel D, containing the optimal combination of Pitavastatin and Irinotecan, was 39.67%. Therefore, it was determined that the brain cancer chip was able to give drug screening results as soon as four days after drug application. In comparing the combinatorial drug treatment in channel D with the individual drug screenings and the untreated GMB spheroid cells, it was found that on day 4, the cell viabilities in the untreated, Pitavastatin treated, and Irinotecan treated spheroids decreased to 95%, 85% and 50%, respectively. Therefore, the combinatorial drug treatment was more effective than those of individual drugs. Furthermore, the cell viability of the untreated GMB spheroids was above 90% on day 7, which confirms the stability of the platform.

Our platform, based on PEGDA microfluidic systems, differs from existing microfluidic platforms, which utilize PDMS microfluidics technology. PEGDA is photo-polymerizable and can be solidified after brief exposure to UV light. Since PDMS suffers from small hydrophobic molecule adsorption and PEGDA blocks non-specific protein adsorption^11^, the use of PEGDA in our approach directed GBM cells to grow into tumor spheroids, creating an *in vitro* 3D microenvironment and suggesting that our platform better mimics the *in vivo* 3D microenvironment. While the normal fabrication of PDMS microfluidic devices takes two days, the fabrication of our hydrogel microfluidic platform takes less than two hours. The photo-polymerization procedure in our platform production requires low UV density and thus eliminates the need for an expensive chrome photomask, which is required to produce the PDMS microfluidic platform. The economical plastic photomask allows for quick modification and flexibility in constructing the PEGDA microfluidic patterns. Furthermore, our platform provides a tunable drug release, which cannot be achieved by existing microfluidic drug testing platforms, including PDMS-based platforms. The tunable drug release capability is the most significant advantage of our microfluidic platform.

The layered structure of the 3D GBM spheroid resulted in a hypoxic center similar to the *in vivo* environment of a GBM tumor mass during the early stages of tumor development, indicating that our hydrogel microfluidic device successfully functions as a brain cancer chip.

In future application, a tiny tissue sample can be collected from a patient by biopsy and cultured in this brain cancer chip. Various drugs can be applied in massive-parallel form. In a week, quick semi-quantitative results can be observed by judging the color of each channel with the naked eye. This chip can also be directly interfaced with custom software to quantify the outcomes and simplify data collection and analysis for clinicians. These potentials make this brain cancer chip a powerful platform for quick preliminary assessments for the efficacy and success of potential drug(s) prior to individual patient administration for personalized medicine. Also, the cross-linking of PEGDA under UV light can efficiently and inexpensively generate microfluidic devices, allowing for widespread application, and is especially promising in developing countries.

## Methods

### Fabrication of the hydrogel microfluidic device

3-(Trimethoxysilyl)propyl methacrylate 98% (TMSPMA) was used to generate an adhesive surface to PEGDA hydrogels on the cover glass (24 × 60 mm cover glasses purchased from Corning Incorporated). Briefly, glass slides were placed in 10% (w/v) NaOH overnight and then washed with distilled water, ethanol 70%, and ethanol 100% repeatedly. After drying at ambient temperature, glass slides were stacked and wetted by 2 ml of TMSPMA before keeping at 70 °C overnight. Finally, TMSPMA-coated glass slides were rinsed with ethanol three times, dried, and stored at room temperature. The top cover glass was drilled with four orifices that were three outlets and one inlet of the chip. The microfluidic patterns were designed by using AutoCAD (Autodesk, Inc.) and then printed on transparent plastics (Fig. 1a) as photomasks (purchased from CADart Washington, USA). The PEGDA brain cancer chip was fabricated by using the soft lithography method shown in Fig. 1a. PEGDA monomer (MW = 750 Daltons, Polysciences, Inc., Warrington, PA) was dissolved in PBS at a concentration of 40% (w/w) with the presence of photoinitiator 2-Hydroxy-4-(2-hydroxyethoxy)-2-methylpropiophenone (PI). After mixing thoroughly, 1 ml PEGDA monomer solution prepared above was pipetted in between two cover glasses for polymerization (top cover glass contained orifices for inlets and outlet, and the bottom cover glass had two spacers). Photo-polymerization was initiated with an Omnicure S2000 (320–500 nm, EXFO, Ontario, Canada) lamp at 100 mW/cm^2^ (measured for 365 nm) at a working distance of 16 cm. After UV exposure, the PEGDA monomer solution was cross-linked into solid PEGDA hydrogel and integrated with the two cover glasses to form a chip. This chip was washed in PBS to remove uncross-linked liquid monomers and PI. Then, adhesive rings were sealed onto the orifices as inlet and outlet reservoirs.

### Optimization of the UV exposure time

Brain cancer chips with different thicknesses were prepared by adjusting the height of spacers between two cover glasses. Nine spacers from 200 μm to 900 μm in height were used and each chip with designed spacer was exposed under the UV light from 20 seconds to 35 seconds to optimize the suitable experimental conditions. PEGDA hydrogel failed to crosslink with insufficient exposure, while the extended exposure resulted in over-cross-linking that blocked the channels and the microwells. The increase of hydrogel layer thickness required longer UV exposure time.

### Stability and leaking test of hydrogel microfluidics

Chicago blue (0.993 mg, 0.001 mmol, Sigma) was dissolved in PBS to a concentration of 5 μM. One ml of Chicago blue solution was injected gradually from an inlet to observe flow in the microchannel. Excess liquid at the outlet was removed gently by pipet. Liquid leakage was confirmed by spread of Chicago blue out of the microchannel.

### Computational modeling

The microfluidic platform was modeled by COMSOL Multiphysics (www.comsol.com) to assess the channel composition. A 3D model was implemented to estimate the chemical-input ratio in different channels and their concentrations in the mixtures. For the multi-physics modeling, we used a modeled geometry with the same dimensions as our PEGDA hydrogel platform. FITC solutions at concentrations of 50 μM and 0 μM were injected into the left and right inlets, respectively. Assuming creeping flow and a stationary solver, the fluid concentration rates in the microwells along the channels were estimated using the incompressible Stokes equations:


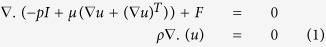


where ρ is fluid density, u is the local velocity, p is the pressure (Pa) and μ is the fluid viscosity. Diffusion constant was set to 2.3 × 10^−10^. A no-slip condition was imposed on the capillary walls, with zero pressure at the outlet, while fluid concentration at inlets were set to 10^−5 ^μM and 0 μM, respectively. Hypothesizing that the flow rates are given and the viscosity is the same everywhere in the hydrogel microfluidics, the concentrations of FITC reduced gradually from the left to the right channels. Channels 1 and 2 were measured at 48 μM, revealing that the ratio of the mixture between two solutions from left and right inlets was 96:4. In channels 3 and 4, 71% of the chemical in the solution mixture came from the solution injected through the left inlet. This ratio continued to decline to 35:65 in channels 5 and 6 and reached the ratio of 4:96 in channels 7 and 8 (see Supplementary Fig. S1). This shows that the chemical composition in individual channels and microwells can be estimated for drug screening, and applied to predict the combinatorial drug treatment.

### Characterization of diffusion

Eosin (0.65 mg, 0.001 mmol, Sigma) was added to 100 ml of PBS to a final concentration of 10 μM. Chicago blue 5-μM and Eosin 10-μM were injected simultaneously into two outer inlets (Fig. 2d). The color appearances in channels change from distinct red (left) to blue (right). For further investigation of the solution gradients in the channels, two 200-μl solutions of 10 μM FITC and PBS were injected concurrently into two outer inlets (Fig. 2e). The fluorescent intensities of FITC in the channels were quantified using ImageJ (National Institutes of Health) and are shown in Fig. 2e.

A 200-μl solution of 10-μM FITC was injected into the center inlet and channels containing fluorescence were observed under an Olympus fluorescence microscope. Time-lapse images were taken at 15 sec, 1 min, 3 min, 5 min, 10 min, 15 min, 20 min and 30 min (Fig. 3a). The fluorescent intensities were quantified using ImageJ (National Institutes of Health). A similar experiment was repeated to investigate the diffusion of DAPI (a low molecular weight molecule) in hydrogel platform. To verify the microfluidics platform’s tunable chemical release, 1 ml solution of FITC 10 μM was injected into a channel (300 μm width) with a microwell (1000 μm diameter). Video was taken by time-lapse imaging by an Olympus fluorescence microscope for 1 min. Time-lapse images were taken at different time points (1 min, 3 min, 5 min and 10 min) to quantify the diffusion of FITC to hydrogel from the microwell. Next, 1 ml PBS was inserted to remove all FITC solution in the microwell. Video and time-lapse images were recorded (Fig. 3c). The fluorescent intensities were quantified using ImageJ (National Institutes of Health).

### Preparation and culture of GBM cells

The U87 cells were cultured in cell culture medium consisting of DMEM supplemented with 10% (v/v) FBS, 100 U/ml penicillin and 100 μg/ml streptomycin. All cells were manipulated under sterile tissue culture hoods and maintained in a 95% air −5% CO_2_ humidified incubator at 37 °C. The U87 cells were plated at a concentration of 5 × 10^6 ^cells/ml in 10-cm diameter tissue culture dishes and were passaged every 3 days at a subculture ratio of 1:4. After trypsinization and centrifugation, the U87 cells were collected and calculated using a hemacytometer (Hausser Scientific, 1483). To test cell capture efficiency in different designs of microchannels and microwells, the cell suspensions were diluted with the cell medium to prepare exact concentrations (2 × 10^6 ^cells/ml) and seeded into the microfluidic device from the center inlet. Cell capture videos were taken by time-lapse imaging using Olympus fluorescence microscope (see Supplementary Video 3). Extra cells in the microchannels were removed by adding 1 ml fresh medium twice. In addition to the microfluidic geometry, the microchannel and microwell dimensions play an important role in achieving high cell capture efficiency.

To optimize a proper cell seeding concentration, a series of cell concentrations of U87 cells (0.01 × 10^6 ^cells/ml, 0.05 × 10^6 ^cells/ml, 0.2 × 10^6 ^cells/ml, 0.5 × 10^6 ^cells/ml and 2 × 10^6 ^cells/ml) were injected (100 μl) into the center inlet. The cells aggregated to form cancer spheroids and were cultured in the microfluidic microwells for 7 days; medium was changed every 24 hours by removing 500 μl of solution from the outlet, and adding 500 μl of fresh growth media into the inlets. Time-lapse images were taken by fluorescence microscope to monitor the cancer spheroid growth. The Live/Dead Cell Viability Assay Kit was performed to measure the U87 cell viability in the microfluidic microwells. The kit, containing calcein AM (2 μg/ml in PBS) and ethidium homodimer (4 μg/ml in PBS) reagents, was prepared as per manufacturer’s instructions. The prepared Live/Dead solution was dropped into the center inlet on the hydrogel microfluidics, and was incubated with cells in the microfluidic microwells for 30 minutes and the images were captured. Green fluorescence was measured due to the calcein AM reaction from viable cells and damaged cell membranes were stained red due to their reaction with ethidium homodimer.

### Combinatorial drug screening

Cancer spheroids were formed and cultured for 7 days. On the first day of dual-drug treatment, Pitavastatin (10 μM, 100 μl) and Irinotecan (100 μM, 100 μl), prepared in Dulbecco’s phosphate buffered saline (PBS), were injected into the two inlets (Fig. 5a), and the hydrogel microfluidic platforms were cultured and maintained in a 95% air-5% CO_2_ humidified incubator at 37 °C. The growth media was exchanged every other day by removing 500 μl of solution from the outlet, and adding 500 μl of fresh growth media into the inlets. Time-lapse images were taken from Olympus fluorescence microscope at day 1, day 4 and day 7 of dual-drug treatment to observe the efficiency of the drugs on cancer spheroids (Fig. 5b). Cells from each microwell were harvested and checked for cell viability on days 1, 4, and 7 after drug administration. Each microchannel was cut from the hydrogel platform and the cells were harvested to check cell viability. Cells were washed twice with PBS and mixed with an equal volume of 0.4% trypan blue for 3 minutes. The unstained (viable) and stained (lysed) cells were counted using a hemacytometer.

### Typan blue staining and quantitative characterization

Trypan blue staining was used to check cell viabilities on day 4 after drug administration in the brain cancer chip. Cells were washed twice with 200 μl PBS injected from inlets. Next, 200 μl of 0.4% trypan blue was added and the chip was incubated for 3 minutes. The unstained (viable) and stained (lysed) cells were observed under Olympus fluorescence microscope.

## Additional Information

**How to cite this article**: Fan, Y. *et al.* Engineering a Brain Cancer Chip for High-throughput Drug Screening. *Sci. Rep.* **6**, 25062; doi: 10.1038/srep25062 (2016).

## Supplementary Material

Supplementary Video 1

Supplementary Video 2

Supplementary Video 3

Supplementary Information
