# Supplementary material for: Engineering a Brain Cancer Chip for High-throughput Drug Screening
Source: Sci Rep. 2016 May 6;6:25062. doi: 10.1038/srep25062 (PMC4858657; doi:10.1038/srep25062)
Supplement: Supplementary Information [file srep25062-s4.pdf]

Supplementary Information

Engineering a Brain Cancer Chip for High-throughput Drug Screening

Yantao Fan[1], Duong Thanh Nguyen[1], Yasemin Akay[1], Feng Xu[2] & Metin Akay[1,*]

[1] Department of Biomedical Engineering, University of Houston, 3605 Cullen Blvd, Room 2027, Houston, TX 77204-5060. Fax: +1 713-743-0226; Tel: +1 832-842-8813

[2] Bioinspired Engineering and Biomechanics Center, Xi'an Jiaotong University, Xi'an 710049, China

[*] Corresponding author: M. AKAY (makay@uh.edu)

1. Supplementary Figure

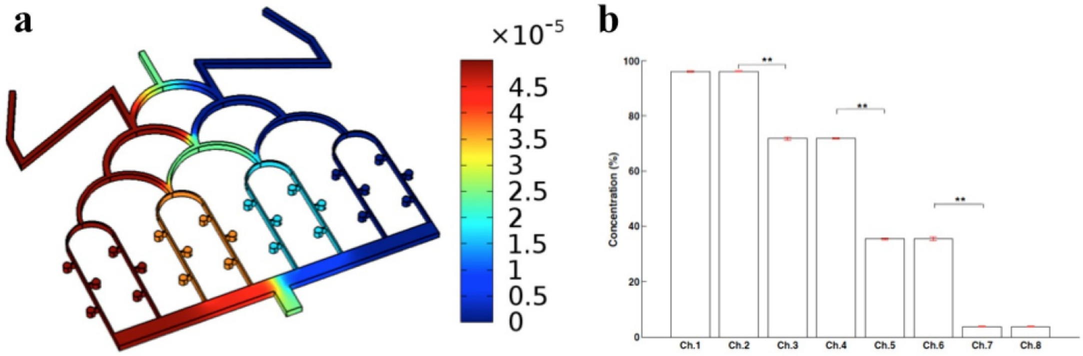

Supplementary Figure S1. Computational modeling of microfluidic platform. (a) Solution containing different chemicals were injected into the inlets and allowed to flow through hydrogel microfluidic chip. The fluid streams were mixed and split repeatedly at intersections to yield mixtures with differing compositions in each microchannel. Two solutions of FITC at different concentrations (10 μM and 0 μM) were tested by introducing them into the left and right inlets, respectively. The microfluidic network generated a concentration gradient. (b) Theoretical concentrations of FITC in microchannels. Microchannels were numbered from left to right. The computational data points are mean values of the concentrations in three microwells of each microchannel. The error bars indicate standard deviations.

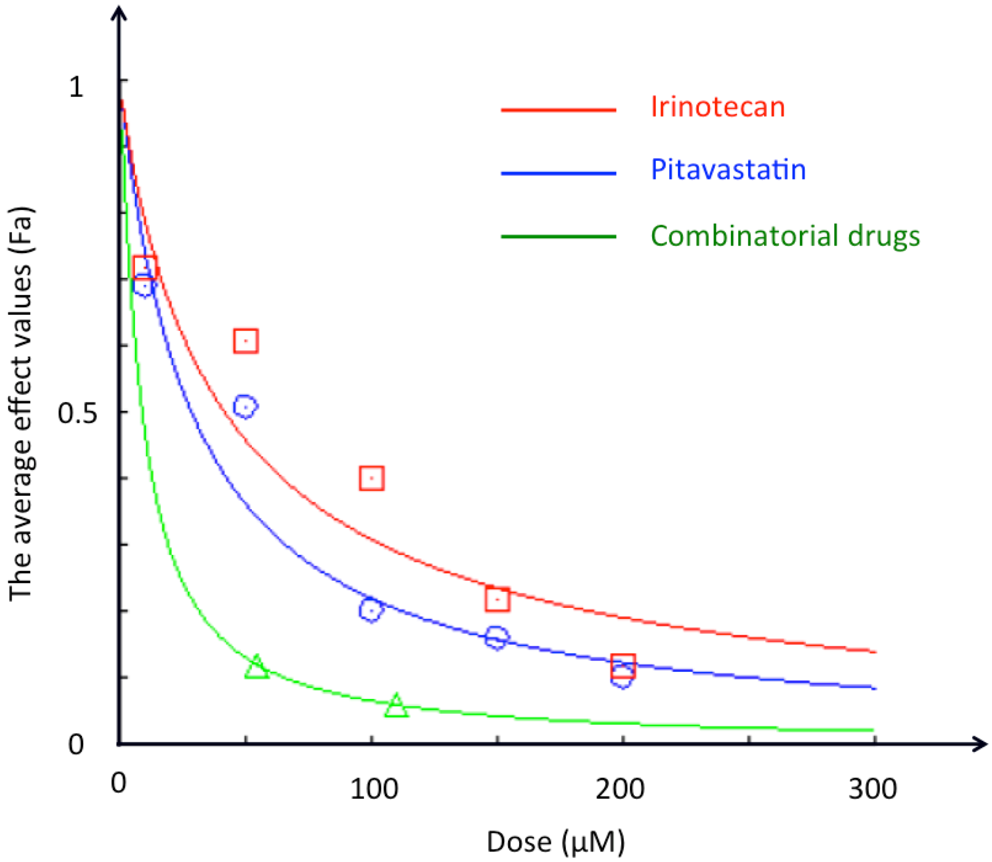

Supplementary Figure S2. The Chou-Talalay (CT) predictive model to study the synergy and antagonism between these two drugs. Red line presented the average effect values of Irinotecan at different doses. Blue line presented the average effect values of Pitavastatin at different doses. Green line showed the average effect values of combinatorial drugs (Pitavastatin and Irinotecan at the ratio 1:10). The CT model suggested that the drug combination is much more effective than individual drugs.

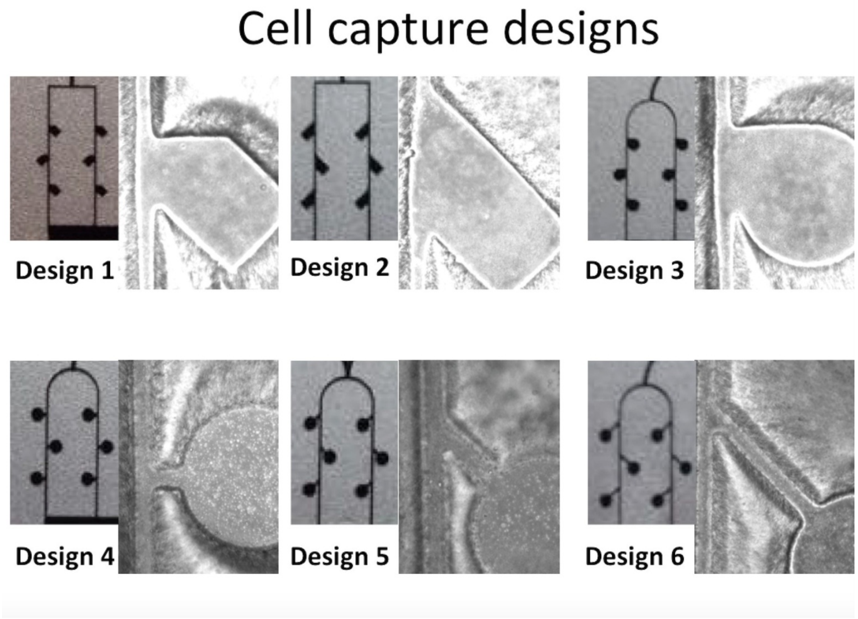

Supplementary Figure S3. Six different designs of microwells and microchannels were fabricated to test cell capture capability. Cells (U87, 2×10^6 cells/ml, 200 µl) were injected into center inlet of each design. Cells that flowed through microchannels and were captured in microwells were recorded with an Olympus fluorescence microscope.

2. Supplementary Video

Supplementary Video 1 (FITC flows into microwell). 1 ml FITC (10 μM) was injected into a 300 μM-wide microchannel. After entering the microwell, FITC diffused into hydrogel surrounding the microwell.

Supplementary Video 2 (PBS flows into microwell to wash FITC). To wash away the FITC in the microwell, 3 ml of PBS was quickly injected into the microchannel. The FITC concentration in microwell reduced to less than the FITC concentration in the surrounding hydrogel. By diffusion, FITC from the hydrogel was released back into the microwell.

Supplementary Video 3 (cell capture). Six different designs of microwells and microchannels were fabricated to test the cell capture capability. Cells (U87, 2×10^6 cells/ml, 200 μl) were injected into the center inlet of each design. Cells flowed through the microchannels and were captured in the microwells which was recorded using an Olympus fluorescence microscope.
